# Unveiling anti-atherosclerotic targets of *Perilla frutescens* through a multi-scale computational framework integrating network pharmacology, single-cell analysis, machine learning, and molecular dynamics

**DOI:** 10.1186/s40643-026-01087-4

**Published:** 2026-06-13

**Authors:** Chenchen Yang, Jianrong Xing, Mengzhu Wang, Wanyi Zhou, Ying Yang, Wenyang Tao

**Affiliations:** Institute of Food Science, Zhejiang Academy of Agricultural Sciences, No.298 Desheng Road, Shangcheng, Hangzhou, 310022 Zhejiang China

**Keywords:** Atherosclerosis, *Perilla frutescens*, Single-cell RNA sequencing, Machine learning, Molecular dynamics

## Abstract

Medicinal plants have long served as important sources of therapeutic agents owing to their diverse bioactive constituents and multi-target pharmacological properties. In particular, plant-derived compounds have attracted increasing attention for the management of chronic inflammatory and metabolic diseases, including atherosclerosis. However, the molecular mechanisms by which medicinal plants modulate the cellular heterogeneity and intercellular communication networks within atherosclerotic plaques remain insufficiently understood. Despite the widespread implementation of lipid-lowering therapy, the persistence of residual inflammatory risk, driven by immunometabolic network dysregulation, remains a cardinal therapeutic challenge in atherosclerosis (AS) management. While *Perilla frutescens* exhibits well-documented anti-inflammatory properties, the precise molecular targeting within the atherosclerotic plaque microenvironment and the regulatory mechanisms governing intercellular communication networks remain poorly elucidated. To address this gap, we established a multi-scale integrative computational framework synergizing network pharmacology, human atherosclerotic plaque single-cell transcriptomic (scRNA-seq) profiling, and ensemble machine learning algorithms (LASSO and random forest) for systematic identification of robust therapeutic targets. Subsequently, molecular docking coupled with 100-ns all-atom molecular dynamics (MD) simulations validated the binding affinity and thermodynamic stability of drug–target complexes. The study successfully analyzed the cellular heterogeneity lineage of plaques and identified a core feature set of 10 genes including HIF1A, PPARG and ITGB1, which specifically mapped the differentiation trajectory of macrophages to foam cells. External validation in an independent cohort demonstrated superior diagnostic performance of this signature (AUC = 0.996). Cellular communication network dissection revealed the foam cell-driven SPP1–ITGB1 signaling axis as a pivotal conduit orchestrating inflammatory crosstalk. Molecular docking demonstrated pronounced binding affinity between luteolin, the principal bioactive constituent of *Perilla frutescens*, and ITGB1 (binding energy: − 8.9 kcal/mol). MD simulations further corroborated the efficacy of luteolin in stabilizing ITGB1 conformation via a "conformational-locking" mechanism (RMSD equilibration within 0.10–0.20 nm), thereby abrogating pathological cell adhesion signaling transduction. Collectively, this study provides a high-resolution molecular atlas of *Perilla frutescens*-mediated AS intervention, systematically elucidating the mechanistic paradigm whereby luteolin attenuates vascular inflammation through targeted disruption of the SPP1–ITGB1 communication axis. These findings underscore the therapeutic targeting of cell adhesion receptors as a translationally promising strategy for mitigating residual inflammatory risk in AS.

**Graphical abstract:**

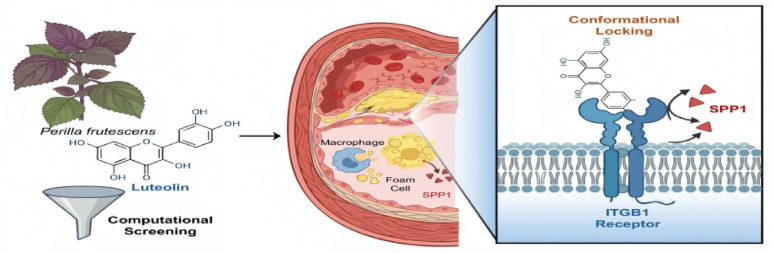

## Introduction

Medicinal plants have been used for centuries across diverse traditional medical systems for the prevention and treatment of various ailments. Historical and ethnopharmacological records indicate that plant derived remedies have played important roles in healthcare practices across different cultures and geographical regions, covering inflammatory disorders, gastrointestinal diseases, respiratory conditions, metabolic abnormalities, infections, and chronic degenerative diseases. These traditional applications provide an important empirical foundation for modern pharmacological investigations and support the continued exploration of medicinal plants as valuable sources of therapeutic agents (Hayta et al. 2014; Meddour and Meddour-Sahar 2015; Petrovska 2012).s

In addition to their traditional therapeutic applications, medicinal and aromatic crops are increasingly recognized as important natural resources because of their nutritional, phytochemical, and pharmacological properties. Representative plant resources such as licorice and isabgol contain diverse bioactive constituents and have been widely discussed in the context of medicinal and aromatic crop utilization. Moreover, nutritional plants such as okra also provide valuable bioactive and nutritional components, further indicating the broad relevance of plant-derived resources in health-related applications (Andualem 2023; Maqbool et al. 2023a, b).

Modern experimental and computational studies have further expanded the scientific understanding of medicinal plants and their bioactive compounds (Abubakar et al. 2024). Plant extracts have shown biological activities against parasitic and microbial pathogens, while medicinal plants used as feed additives have been reported to influence productive and immunological performance (Dalal et al. 2023). More importantly, herbal flavonoids and other plant-derived compounds have been investigated through pharmacological networking and in vitro validation, supporting the concept that medicinal plants may exert therapeutic effects through multi-component, multi-target, and multi-pathway mechanisms (Hegazy et al. 2023; Yasin et al. 2025).

In the context of cardiovascular diseases, medicinal plants have attracted increasing attention as potential complementary therapeutic resources for atherosclerosis. Atherosclerosis is closely associated with lipid accumulation, oxidative stress, endothelial dysfunction, immune cell infiltration, and chronic vascular inflammation, all of which may be modulated by plant-derived bioactive compounds. Therefore, medicinal plants may provide promising candidates for regulating the complex pathological networks involved in atherosclerotic plaque formation and progression (Kirichenko et al. 2020). Atherosclerosis (AS) represents a chronic progressive pathological condition fundamentally characterized by arterial intimal lipid accumulation and maladaptive immunological responses. Despite the demonstrated efficacy of lipid-lowering pharmacotherapy, particularly statins and proprotein convertase subtilisin/kexin type 9 (PCSK9) inhibitors, substantial residual inflammatory risk (RIR) persists, perpetuating plaque progression and precipitating acute cardiovascular events (Di Muro et al. 2025). Emerging mechanistic investigations have revealed that AS is orchestrated by intricate immunometabolic networks, wherein dysregulated crosstalk between lipid metabolism and inflammatory signaling establishes a homeostatic pathological architecture exhibiting pronounced resistance to monotherapeutic interventions (Z. Wang et al. 2025a, b). Consequently, therapeutic paradigms necessitate a strategic transition from linear pathway inhibition toward systemic network perturbation to enable synergistic modulation of multiple pivotal nodes within the atherosclerotic plaque microenvironment.

Botanical therapeutics, by virtue of their multi-component synergistic architecture, constitute a promising reservoir of bioactive compounds for network-based modulation. *Perilla frutescens* frutescens seeds, a quintessential functional food with medicinal attributes, harbor an abundance of pleiotropic bioactive constituents encompassing α-linolenic acid, phytosterols, and flavonoids such as luteolin. While prior phenotypic investigations have established the therapeutic efficacy of *Perilla frutescens* in ameliorating dyslipidemia and suppressing vascular inflammation, the underlying molecular mechanisms remain incompletely elucidated (Wu et al. 2023). Specifically, the precise cellular targeting and mechanistic execution of vasculoprotective effects by these bioactive constituents within the heterogeneous cellular landscape of atherosclerotic plaques remain undefined.

Conventional systems pharmacology predominantly relies on bulk tissue transcriptomic profiling, the inherent methodological constraints of which substantially impede mechanistic interrogation at cellular resolution. This aggregate approach fundamentally obscures the spatiotemporal heterogeneity intrinsic to atherosclerotic plaques, precluding effective discrimination between pathogenic cellular subsets (e.g., specific foam cell phenotypes) and bystander populations (Xiong et al. 2023). Moreover, plaque destabilization is orchestrated through intricate intercellular communication networks, including ligand–receptor interactions governing cell adhesion and migration, the architectural delineation of which remains intractable without single-cell resolution. Consequently, critical knowledge gaps persist regarding which cellular differentiation trajectories are targeted by *Perilla frutescens* and the mechanisms through which it disrupts pathological intercellular crosstalk driving AS progression.

To address these knowledge deficits, we established an integrative computational framework synergizing single-cell RNA sequencing (scRNA-seq) with machine learning (ML) algorithms. We employed an ensemble feature selection strategy coupling least absolute shrinkage and selection operator (LASSO) regression with random forest (RF) algorithms to rigorously identify robust core targets from high-dimensional single-cell datasets, thereby mitigating transcriptomic noise and overfitting artifacts. Furthermore, molecular docking coupled with molecular dynamics (MD) simulations validated the thermodynamic stability and conformational dynamics of predicted drug–target complexes under quasi-physiological solvation conditions.

Leveraging this strategy, we successfully deconvoluted the cellular heterogeneity landscape of human atherosclerotic plaques and identified a core genetic signature encompassing HIF1A, PPARG, and ITGB1. Critically, our investigation revealed high-affinity binding between luteolin, the principal bioactive constituent of *Perilla frutescens*, and integrin β1 (ITGB1), whereby conformational stabilization via a "conformational-locking" mechanism abrogates the SPP1–ITGB1 signaling axis, which serves as a pivotal conduit mediating foam cell adhesion and inflammatory activation. Collectively, these findings delineate a high-resolution molecular cartography of *Perilla frutescens*-mediated AS intervention, underscoring the translational potential of targeting cell adhesion receptors for mitigating residual vascular inflammation.

## Materials and methods

### Screening of bioactive constituents and target prediction for *Perilla frutescens*

Chemical constituents of *Perilla frutescens* were systematically retrieved from the Traditional Chinese Medicine Systems Pharmacology (TCMSP) database (https://tcmsp-e.com) using “*Perilla frutescens*” as the query keyword (Ru et al. 2014). Candidate bioactive constituents were filtered based on conventional absorption, distribution, metabolism, and excretion (ADME) criteria, with selection thresholds established at oral bioavailability (OB) ≥ 30% and drug-likeness (DL) ≥ 0.18. Redundant entries were eliminated, and compound identifiers were standardized to facilitate downstream target prediction analyses. Putative molecular targets of candidate constituents were predicted using SwissTargetPrediction, Similarity Ensemble Approach (SEA), and SuperPred, with species parameters restricted to *Homo sapiens*. Prediction outputs from these platforms were subsequently merged, deduplicated, and subjected to standardized mapping via the UniProt database (https://www.uniprot.org) for uniform conversion to gene symbol nomenclature (Islam et al. 2025; Rasheed et al. 2026).

AS-associated disease genes were retrieved from GeneCards (https://www.genecards.org), Online Mendelian Inheritance in Man (OMIM; https://www.omim.org), and DisGeNET (v7.0; https://www.disgenet.org) using "Atherosclerosis" as the query term. Selection criteria comprised: relevance score ≥ 10 for GeneCards entries, gene-disease association score (Score_gda) ≥ 0.10 for DisGeNET entries, and explicitly annotated AS-associated genes from OMIM. Disease-associated genes retrieved from these three repositories were consolidated and deduplicated to establish a comprehensive disease gene set.

Intersection analysis between the *Perilla frutescens* candidate target gene set and the AS disease gene set was subsequently performed to identify putative therapeutic targets. Set intersection operations and visualization were executed in R software (v4.3.2) using the ggVennDiagram package (v1.2.2) for Venn diagram construction. The resultant intersecting targets were further subjected to protein–protein interaction (PPI) network construction, functional enrichment analysis, and integrative prioritization with single-cell transcriptomic differential expression profiles.

### PPI network construction, GO and KEGG enrichment analyses

The PPI network of intersecting targets was constructed using the Search Tool for the Retrieval of Interacting Genes/Proteins (STRING) database (v11.5; https://string-db.org) (Szklarczyk et al. 2021). Analytical parameters were configured as follows: species restriction to Homo sapiens and minimum interaction confidence threshold set to high confidence (combined score > 0.7). PPI outputs were subsequently imported into Cytoscape (v3.9.1) for network visualization and topological characterization, with isolated nodes excluded to generate a connected network for downstream analyses (Shannon et al. 2003). The combination of PPI network construction and topological analysis represents a commonly used network pharmacology strategy for identifying hub targets and functional interaction modules of herbal medicines (Wang et al. 2022).

Functional enrichment analyses were conducted in the R environment utilizing the clusterProfiler package (v4.10.0), with gene annotation information sourced from the org.Hs.eg.db database (v3.18.0) (Wu et al. 2021). To enhance annotation consistency and analytical robustness, gene symbols were initially converted to Entrez Gene IDs, followed by Gene Ontology (GO) enrichment analysis across three ontological dimensions, including biological process (BP), cellular component (CC), and molecular function (MF), as well as Kyoto Encyclopedia of Genes and Genomes (KEGG) pathway enrichment analysis. Multiple hypothesis testing correction was implemented via the Benjamini–Hochberg (BH) procedure, with the significance threshold established at false discovery rate (FDR, p.adjust) < 0.05. Enrichment outputs were visualized as bubble plots to systematically delineate the core biological processes and pivotal signaling cascades engaged by *Perilla frutescens* putative therapeutic targets (Zhang 2023).

### Single-cell transcriptomic data acquisition and preprocessing

The human atherosclerotic plaque single-cell transcriptomic dataset GSE159677 was retrieved from the Gene Expression Omnibus (GEO) repository, with extraction of publicly available gene–cell expression matrices and associated metadata files (Alsaigh et al. 2022). All computational analyses were executed in the R environment (v4.3.2), with single-cell data preprocessing and downstream analytical workflows primarily implemented using the Seurat package (v4.3.0). Seurat objects were constructed from expression matrices, followed by implementation of stringent quality control (QC) procedures to eliminate low-quality cells and mitigate potential technical noise artifacts (Hao et al. 2021).

Subsequently, doublet artifacts were identified and excluded using DoubletFinder (v2.0.3). Parametric configurations were determined based on sample-specific cell counts and standardized protocols, with anticipated doublet rates established according to 10 × Genomics empirical recommendations and optimal parameter combinations refined through pK value sweep optimization. QC-filtered data underwent normalization via the Seurat NormalizeData function, followed by identification of highly variable genes (HVGs) using the FindVariableFeatures function. Data scaling and standardization were subsequently performed using the ScaleData function, with regression of technical covariates (including sequencing depth and mitochondrial gene fraction) to mitigate batch-associated technical variation. Principal component analysis (PCA) was executed via the RunPCA function, with optimal dimensionality for downstream analyses determined through integrative assessment of ElbowPlot visualization, JackStraw permutation testing, and cumulative variance explained metrics. To mitigate potential confounding effects of batch variation on cellular clustering architecture, multi-sample integration was performed using the Seurat integration workflow, with subsequent construction of k-nearest neighbor (kNN) graphs and execution of clustering algorithms within the integrated low-dimensional embedding space.

Cell type annotation was manually assigned based on canonical marker gene expression profiles, with cross-validation performed through visualization modalities including FeaturePlot and DotPlot representations. Upon completion of cell type annotation, differentially expressed genes (DEGs) were identified using the FindMarkers function (default: Wilcoxon rank-sum test), with multiple testing correction implemented via the Benjamini–Hochberg (BH) procedure. DEG selection criteria comprised FDR (p.adjust) < 0.05, with the resultant DEG signature subjected to integrative cross-validation against network pharmacology-derived candidate targets for identification of core therapeutic nodes.

### Machine learning-based feature selection for disease state classification

Feature selection was performed using two complementary machine learning algorithms, namely least absolute shrinkage and selection operator (LASSO) regression and random forest (RF). To circumvent pseudo-replication artifacts inherent to single-cell data structures, pre-modeling aggregation of single-cell expression profiles was performed using a pseudo-bulk strategy across “sample × cell type” dimensions, yielding gene expression matrices with biological samples as statistical units. Disease state labels were derived from clinical stratification metadata accompanying the GEO dataset. Training–validation partitioning was executed at the sample level, ensuring mutually exclusive sample allocation to preclude data leakage artifacts.

LASSO regression modeling was implemented using the glmnet package (v4.1–8), with disease state configured as a binary outcome variable and model family specified as binomial. Following feature standardization, optimal regularization parameter λ was determined via tenfold cross-validation, with lambda.1se adopted as the penalty coefficient. Genes exhibiting non-zero regression coefficients at the selected λ value constituted the LASSO-derived feature gene set. Random forest classification models were trained using the randomForest package (v4.7–1.1), with computation of variable importance metrics for individual features. Given potential selection bias inherent to Gini index-derived variable importance, permutation importance was adopted as the primary evaluation metric. Intersection analysis between the LASSO-derived feature set and the top 20 genes ranked by random forest variable importance yielded the core feature gene signature. In instances of insufficient intersection cardinality, the union of both gene sets was employed, with subsequent refinement through external validation and model performance assessment. Candidate feature genes were ranked in descending order of composite importance scores, with the top 20 genes prioritized for downstream analyses.

### Biological weighted expression score (WES) validation

To quantitatively assess the transcriptional activity of candidate genes within key cellular subsets, gene set module scores and weighted WES were computed within the Seurat analytical framework. Initially, module scores for candidate gene sets were calculated using the Seurat AddModuleScore function to quantify relative transcriptional intensity at single-cell resolution, with background gene set normalization correction.

Building upon this foundation, a WES was formulated to integrate both expression abundance and detection frequency within specific cellular subsets:

$$ \begin{aligned} WES = & Average\quad expression \\ & \quad \times Detection\quad rate(Pct.{\mathrm{Exp}}) \\ \end{aligned} $$

here Average Expression denotes the mean normalized expression level within the target cellular subset (computed via the Seurat AverageExpression function from LogNormalize-standardized expression matrices), while Detection Rate represents the detection frequency (pct.exp, defined as the proportion of cells exhibiting expression values > 0). WES values were computed for each candidate gene across individual cellular subsets and ranked in descending order. Subsequently, top-ranked WES genes were subjected to intersection analysis with machine learning-derived feature signatures, with the top 10 WES-ranked intersecting genes designated as core therapeutic targets.

### Spatial and dynamic expression validation of core targets at single-cell resolution

To assess the spatial distribution architecture of core candidate targets across cellular subsets, gene expression was projected onto uniform manifold approximation and projection (UMAP) low-dimensional embeddings using the Seurat FeaturePlot function, with expression heterogeneity across cell types and subpopulations visualized via DotPlot and VlnPlot representations. Subsequently, myeloid lineage cells, encompassing monocytes, macrophages, and foam cell subsets, were subjected to pseudotemporal trajectory reconstruction analysis. Trajectory inference was performed using the Monocle3 package (v1.3.1) (Cao et al. 2019). Trajectory root cells were designated based on cellular subsets exhibiting elevated expression of early macrophage markers (e.g., LST1, FCER1G), with pseudotemporal ordering established via root cell specification through the order_cells() function. Expression dynamics of core targets along the pseudotemporal axis were visualized and subjected to trend-fitting analyses to characterize their transcriptional trajectories during macrophage-to-foam cell differentiation.

### Clinical diagnostic performance validation (ROC analysis)

To evaluate the clinical diagnostic performance of core candidate targets, an independent external validation dataset GSE100927 (n = 104) was retrieved from the GEO repository. Atherosclerotic pathological specimens and healthy controls were extracted based on original sample annotations to establish binary classification labels. Expression data underwent preprocessing and standardization workflows: for microarray platforms, sequential application of background correction, quantile normalization, and log₂ transformation was performed; in instances of multiple probe-to-gene mappings, probe expression values were aggregated to the gene level via median (or mean) summarization to construct gene expression matrices. Subsequently, expression profiles of core genes within the validation cohort were extracted for downstream model construction and performance assessment.

Multivariable logistic regression modeling was performed based on core gene expression profiles, with receiver operating characteristic (ROC) curves generated using the pROC package (v1.18.5). Area under the curve (AUC) metrics and corresponding 95% confidence intervals (CIs) were computed via the DeLong method. To mitigate overfitting-associated performance inflation bias, tenfold cross-validation was implemented within the external validation cohort, with computation of mean AUC values and assessment of model discriminatory capacity across clinical stratifications.

### Molecular docking

Three-dimensional structures of core target proteins were retrieved from the Research Collaboratory for Structural Bioinformatics Protein Data Bank (RCSB PDB), with selection criteria comprising: Homo sapiens species restriction, crystallographic resolution ≤ 2.5 Å, and preferential selection of structures harboring co-crystallized ligands. In instances of missing residues or incomplete side-chain geometries, structural refinement was performed using PDBFixer (v1.9) for residue reconstruction and conformational optimization. Receptor proteins underwent processing in PyMOL (v2.5.5) for removal of redundant water molecules while preserving critical metal ions and cofactors, followed by polar hydrogen addition, Gasteiger partial charge assignment, and PDBQT format conversion using AutoDockTools (ADT, v1.5.7) (Eberhardt et al. 2021). Ligand molecular structures were retrieved from the PubChem database, with three-dimensional conformer generation and geometric energy minimization performed using Open Babel (v3.1.1).

Molecular docking simulations were executed using AutoDock Vina (v1.2.5), with docking grid centers defined based on co-crystallized ligand coordinates or computationally predicted active site pocket topologies. Docking parameters were configured as follows: exhaustiveness = 32, num_modes = 10, energy_range = 3. Docking poses were ranked according to the Vina scoring function, with the lowest-energy conformation selected for protein–ligand interaction analysis and visualization in PyMOL.

### MD simulations

MD simulations were executed using the GROMACS software package (v2024.3) (Abraham et al. 2015). Ligand partial charges were derived via restrained electrostatic potential (RESP/RESP2) fitting of ORCA-computed wavefunctions using the Multiwfn program, with force field topology files generated via the Sobtop utility based on the general AMBER force field (GAFF) (Lu & Chen 2012). Protein parameterization employed the CHARMM36 force field, with explicit solvation maintained throughout via the TIP3P water model. Protein–ligand complexes were positioned within rhombic dodecahedral simulation boxes, with a minimum solute-to-box boundary distance of 1.2 nm. Following explicit solvation, Na⁺ and Cl⁻ counterions were introduced to achieve charge neutralization, with ionic strength adjusted to 0.15 M to recapitulate physiological salinity.

Systems underwent initial energy minimization via the steepest descent algorithm. Subsequently, equilibration simulations were performed sequentially under NVT ensemble conditions (100 ps, velocity-rescaling temperature coupling) and NPT ensemble conditions (100 ps, Parrinello–Rahman pressure coupling), with positional restraints of 1000 kJ·mol^−1^·nm^−2^ applied to protein backbone and ligand heavy atoms. Production-phase simulations were conducted with removal of all positional restraints, employing a 2-fs integration timestep over 100 ns. All hydrogen-containing covalent bonds were constrained via the linear constraint solver (LINCS) algorithm, with long-range electrostatic interactions computed using the particle mesh Ewald (PME) method; van der Waals and short-range electrostatic cutoff radii were uniformly set to 1.0 nm, with neighbor list updates performed every 20 timesteps. Post-trajectory analyses encompassed temporal evolution of root mean square deviation (RMSD), root mean square fluctuation (RMSF), radius of gyration (Rg), and protein–ligand interfacial hydrogen bond occupancy, enabling systematic assessment of complex conformational stability and binding interface persistence.

#### Statistical analysis

Statistical analyses were performed in R. Intergroup comparisons were conducted using two-sample t-tests or Wilcoxon rank-sum tests, with multiple testing correction implemented via the Benjamini–Hochberg procedure (FDR threshold: *p.adjust* < 0.05). ROC analyses employed the pROC package for computation of AUC values and 95% confidence intervals via the DeLong method. Unless otherwise specified, all statistical tests were two-tailed, with *P* < 0.05 considered statistically significant.

## Results

### Screening of bioactive constituents and identification of putative therapeutic targets in *Perilla frutescens*

A network pharmacology approach was employed to systematically dissect the pharmacodynamic material basis underlying the therapeutic efficacy of Perilla frutescens. Initially, 16 core bioactive constituents exhibiting favorable pharmacokinetic properties were identified from *Perilla frutescens* using ADME filtering criteria of oral bioavailability (OB) ≥ 30% and DL ≥ 0.18 within the TCMSP database framework. As illustrated in Table 1, these constituents collectively exhibited elevated bioavailability potential and favorable drug-like structural attributes. Notably, luteolin (MOL000006), a flavonoid constituent prioritized for downstream investigation, demonstrated balanced pharmacokinetic parameters indicative of favorable membrane permeability and intestinal absorption capacity. Remarkably, phytosterol constituents including β-sitosterol (MOL000358) and stigmasterol (MOL000449) exhibited OB values exceeding 36%, with maximal values reaching 43.83%, underscoring the pivotal contribution of lipophilic compounds to *Perilla frutescens* pharmacological repertoire.

**Table 1:** Physicochemical properties and ADME evaluation metrics of candidate active compounds in *Perilla frutescens*

Mol ID	Molecule name	MW	AlogP	OB (%)	DL
MOL000006	Luteolin	286.25	2.07	36.16	0.25
MOL000358	Beta-sitosterol	414.79	8.08	36.91	0.75
MOL000449	Stigmasterol	412.77	7.64	43.83	0.76
MOL000953	Cholesterol	386.73	7.38	37.87	0.68
MOL001439	Arachidonic acid	304.52	6.41	45.57	0.20
MOL002773	Beta-carotene	536.96	12.00	37.18	0.58
MOL004355	Spinasterol	412.77	7.64	42.98	0.76
MOL005030	Gondoic acid	310.58	7.75	30.70	0.20
MOL005043	Campest-5-en-3beta-ol	400.76	7.63	37.58	0.71
MOL005481	2,6,10,14,18-pentamethylicosa-2,6,10,14,18-pentaene	342.67	9.51	33.40	0.24
MOL007449	24-methylidenelophenol	412.77	7.75	44.19	0.75
MOL009653	Cycloeucalenol	426.80	7.59	39.73	0.79
MOL009681	Obtusifoliol	426.80	8.15	42.55	0.76
MOL012888	Citrostadienol	426.80	8.15	43.28	0.79
MOL012891	(2E,4E,6E)-icosa-2,4,6-trienoic acid	306.54	7.28	41.64	0.20
MOL012893	(E)-(4-methylbenzylidene)-(4-phenyltriazol-1-yl)amine	262.34	4.09	57.87	0.19

Subsequently, the integration of multiple target prediction platforms, namely SwissTargetPrediction, SEA, and SuperPred, followed by standardized annotation, yielded 695 putative protein targets potentially interacting with the aforementioned bioactive constituents. Concurrently, systematic interrogation of GeneCards, OMIM, and DisGeNET repositories established an AS -associated target compendium comprising 2,444 genes.

**Fig. 1:**
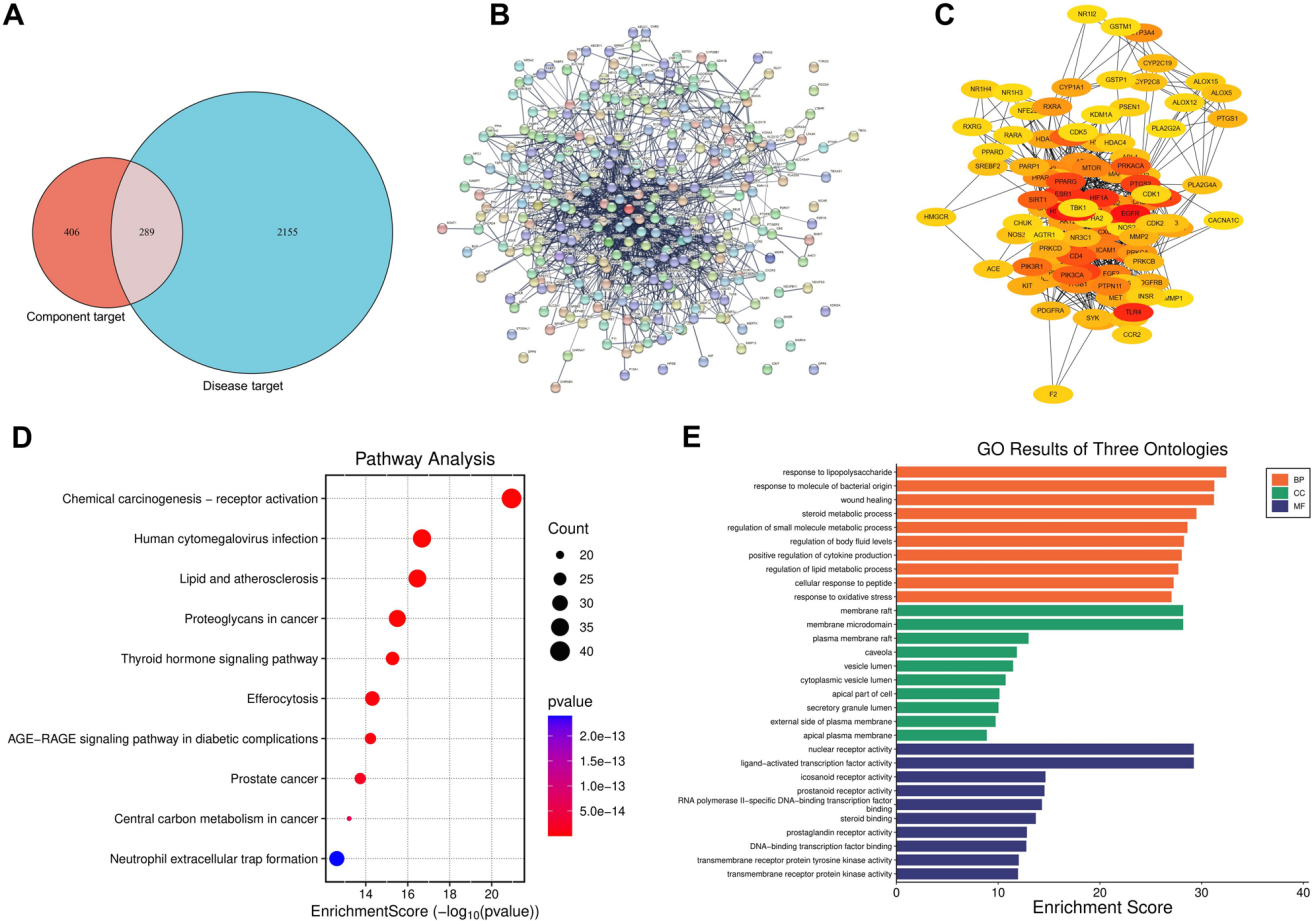
Network pharmacology analysis of *Perilla frutescens* seeds against atherosclerosis. **A** Venn diagram of intersecting targets. **B** PPI network construction. **C** Screening of the core target sub-network. **D** KEGG pathway enrichment. **E** GO functional annotation (BP, CC, and MF)

Venn diagram mapping via the ggVennDiagram package revealed substantial intersection between *Perilla frutescens* candidate targets and the AS disease gene set, yielding 289 overlapping genes (Fig. 1A). These overlapping genes constituted the candidate therapeutic target set for *Perilla frutescens*-mediated AS intervention, providing a critical genetic foundation for elucidating molecular connectivity between botanical bioactive constituents and disease pathophysiology. To further dissect synergistic interaction architectures among candidate targets, the 289 overlapping genes were imported into the STRING database (v11.5) for high-confidence protein–protein interaction (PPI) network construction. Network topology visualization (Fig. 1B) revealed characteristic scale-free network architecture, with dense physical and functional interconnectivity among nodes corroborating the “multi-component–multi-target–multi-pathway” synergistic regulatory paradigm of *Perilla frutescens*. Furthermore, quantitative topological characterization via Cytoscape, incorporating weighted ranking of topological metrics including maximal clique centrality (MCC) and node degree, identified a core functional module comprising highly connected hub nodes (Fig. 1C). These topologically central hub genes exert pivotal regulatory roles within *Perilla frutescens*-mediated modulation of atherosclerotic pathological networks.

### Gene enrichment analysis

To systematically elucidate the biological functionalities of the 289 intersecting targets, multidimensional GO functional enrichment and KEGG pathway enrichment analyses were performed. GO enrichment analyses revealed the molecular and cellular functional landscape underlying the putative therapeutic effects of *Perilla frutescens* (Fig. 1D). Within the biological process (BP) ontological dimension, target genes exhibited significant enrichment in immune defense responses to lipopolysaccharide (LPS) and bacterial-derived molecular patterns, with pronounced associations to wound healing and inflammatory response modulation. This enrichment architecture suggested that *Perilla frutescens* may ameliorate vascular endothelial chronic injury and repair dysregulation through attenuation of pathogen-associated molecular pattern (PAMP)-triggered inflammatory cascades. Additionally, pronounced enrichment of steroid and lipid metabolism-related terms suggested that *Perilla frutescens* may counteract atherosclerotic lipid accumulation pathology through restoration of lipid metabolic homeostasis.

Within the cellular component (CC) ontology, target genes predominantly localized to subcellular structures including membrane rafts, membrane microdomains, and vesicle lumina. Given the pivotal role of membrane rafts in receptor clustering and signaling complex assembly, this enrichment profile indicated that *Perilla frutescens* bioactive constituents may exert vasculoprotective effects through modulation of membrane-associated signal transduction platforms. Within the molecular function (MF) dimension, target genes demonstrated significant enrichment in nuclear receptor activity, ligand-activated transcription factor activity, and eicosanoid receptor activity. This functional landscape aligned closely with the established regulatory roles of nuclear receptors (e.g., PPARs) in governing lipid metabolism and anti-inflammatory transcriptional programs, suggesting transcriptional-level intervention in AS-associated pathological networks by *Perilla frutescens*.

KEGG pathway enrichment analysis further corroborated the mechanistic connectivity between candidate targets and atherosclerotic pathophysiology at the signaling cascade level (Fig. 1E). The “Lipid and atherosclerosis” pathway exhibited the most pronounced enrichment score and maximal gene ratio, indicating non-random distribution of intersecting target genes with specific convergence upon core atherosclerotic pathological modules. Beyond this primary pathway, target genes demonstrated substantial enrichment in pathological processes encompassing efferocytosis, advanced glycation end product–receptor for AGE (AGE–RAGE) signaling axis, and neutrophil extracellular trap (NET) formation. This multi-pathway enrichment architecture suggested vasculoprotective efficacy of *Perilla frutescens* through multidimensional synergistic mechanisms: (i) enhancement of macrophage-mediated intraplaque apoptotic cell clearance, mitigating necrotic core formation; (ii) antagonism of AGE-induced oxidative injury; and (iii) suppression of NET-mediated immunothrombotic responses. Additionally, while certain enrichment terms pertained to chemical carcinogenesis and proteoglycan-associated pathways, these signaling modules likely reflect aberrant vascular smooth muscle cell (VSMC) proliferation and pathological extracellular matrix (ECM) remodeling within the atherosclerotic context. Collectively, GO and KEGG enrichment analyses indicated that *Perilla frutescens* bioactive constituents may attenuate atherosclerotic plaque progression and destabilization through modulation of multiple signaling networks governing cellular proliferation and vascular microenvironmental remodeling.

### Single-cell transcriptomic atlas construction and plaque cellular heterogeneity delineation

**Fig. 2:**
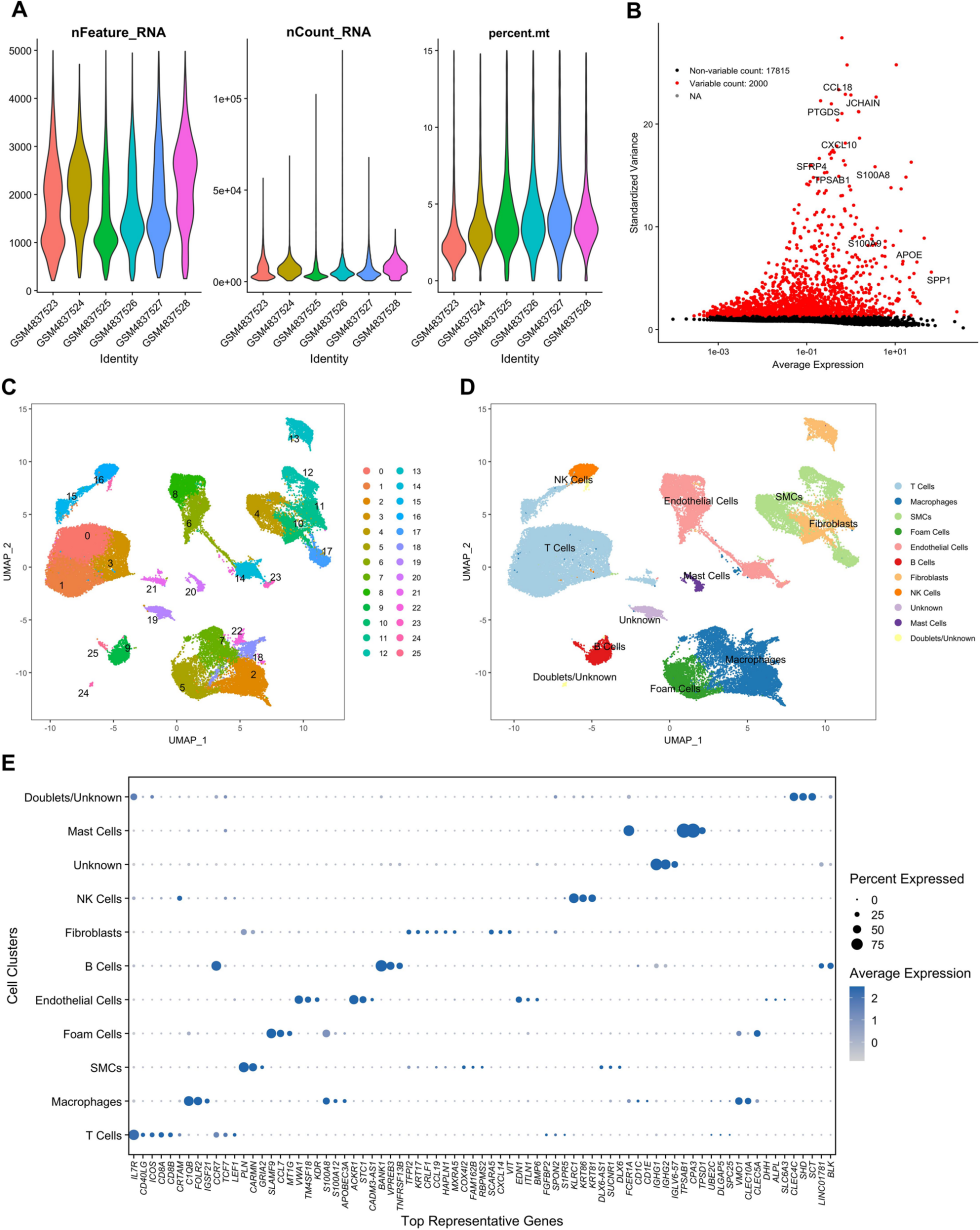
Quality control, clustering, and cell type annotation of scRNA-seq data. **A** Quality control metrics of samples. **B** Identification of highly variable genes. **C** UMAP projection of cell clusters. **D** Annotation of major cell types. **E** Dot plot of marker gene expression

High-resolution single-cell transcriptomic (scRNA-seq) profiling was performed using the GSE159677 dataset, with stringent QC procedures implemented to eliminate low-quality cells and technical noise artifacts. QC metrics revealed uniform distributions of detected gene counts (nFeature_RNA) across all samples (GSM4837523–GSM4837528), with mitochondrial gene expression fractions consistently maintained below 10% (Fig. 2A), ensuring downstream analyses were predicated upon high-fidelity transcriptomic data. Among the 2,000 HVGs identified, CCL18, APOE, SPP1, and S100A8/A9 exhibited maximal expression variability (Fig. 2B). These HVGs predominantly encompassed chemotactic signaling, lipid trafficking, and inflammatory response mediators, indicating pronounced cellular heterogeneity associated with lipid metabolic dysregulation and robust inflammatory activation within the plaque microenvironment.

Subsequently, UMAP nonlinear dimensionality reduction projected the single-cell transcriptomic landscape onto two-dimensional embeddings, with unsupervised clustering algorithms resolving 26 discrete cellular clusters (Fig. 2C). Based on canonical marker gene expression profiles, 11 major cellular lineages were annotated (Fig. 2D), encompassing structural vascular wall constituents, such as endothelial cells, smooth muscle cells (SMCs), and fibroblasts, alongside immune infiltrates including T cells, B cells, natural killer (NK) cells, and mast cells. Critically, higher-resolution dissection of myeloid lineage cells was achieved, enabling unambiguous discrimination of foam cells from classical macrophage populations. Foam cells occupied a discrete clustering topology within UMAP embedding space, indicating substantial transcriptional-level phenotypic reprogramming.

To validate cell type annotation fidelity and characterize lineage-specific molecular signatures, expression distribution patterns of canonical marker genes were visualized via dot plot representation (Fig. 2E). Results revealed distinct and lineage-specific marker gene expression signatures: T cells exhibited selective enrichment of IL7R and CD3D, endothelial cells demonstrated elevated VWF and PECAM1 expression, while smooth muscle cells displayed characteristic ACTA2 and MYH11 upregulation. Critically, foam cells retained myeloid lineage markers (e.g., CD68) while exhibiting pronounced upregulation of inflammation-associated genes including S100A8, S100A12, and MT1G. Given the established roles of these genes in inflammatory signal amplification, metal ion homeostasis regulation, and oxidative stress responses, this differential expression signature indicated that foam cells, following excessive lipid internalization, existed in a pathologically activated state characterized by elevated oxidative stress burden and sustained inflammatory activation.

### Identification of core therapeutic targets via machine learning and clinical validation

**Fig. 3:**
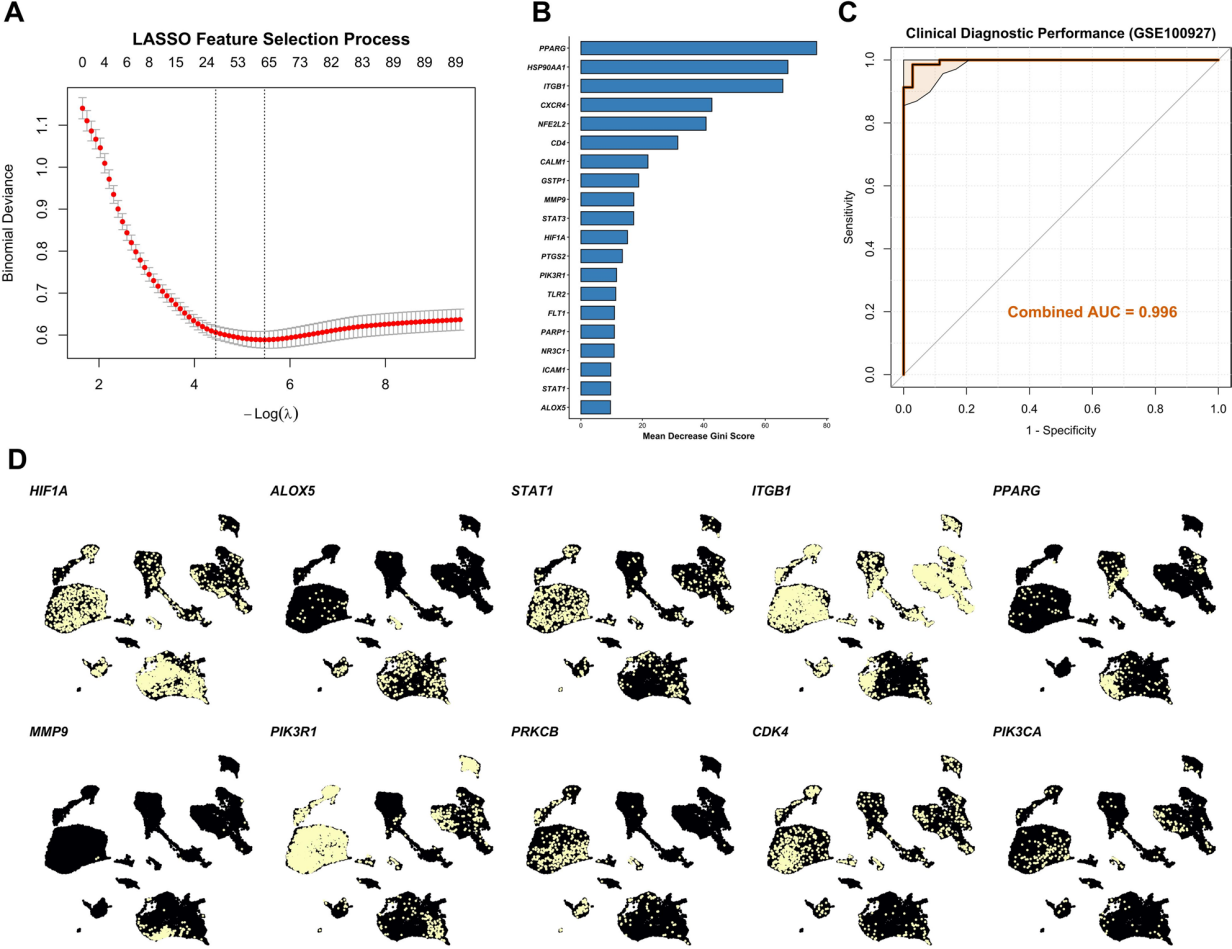
Machine learning screening and validation of core targets. **A** LASSO feature selection process. **B** Variable importance scores from random forest analysis. **C** ROC diagnostic performance analysis in the external dataset. **D** Expression distribution of core targets in the single-cell landscape. The UMAP coordinates and cluster identities in panel D are consistent with the annotated cell populations shown in Fig. 2D. The corresponding clusters include foam cells, macrophages, monocytes, endothelial cells, smooth muscle cells, fibroblasts, T cells, B cells, NK cells, and mast cells

To precisely identify hub genes exhibiting potential clinical diagnostic utility and disease relevance within the intersecting gene set, an integrative feature dimensionality reduction strategy synergizing statistical and machine learning methodologies was implemented. Initially, feature selection was performed via LASSO regression, with regularization parameter λ optimized through tenfold cross-validation. At the λ value corresponding to binomial deviance minimization, redundant variables were effectively eliminated, thereby mitigating multicollinearity-induced compromise of parameter estimation robustness (Fig. 3A). Concurrently, random forest (RF) modeling assessed individual gene contributions to disease state classification from a nonlinear perspective, with variable importance ranking identifying high-priority feature genes including PPARG, ITGB1, MMP9, and ALOX5 (Fig. 3B). This dual-algorithm integrative framework implemented cross-validation of candidate features under divergent modeling assumptions, substantially enhancing feature selection robustness.

Subsequently, intersection analysis between machine learning-derived feature genes and top-ranked WES genes at single-cell resolution yielded 10 core therapeutic targets (Table 2): HIF1A, ALOX5, STAT1, ITGB1, PPARG, MMP9, PIK3R1, PRKCB, CDK4, and PIK3CA. To assess the translational potential of this core target signature, multivariable logistic regression diagnostic modeling was performed within the independent external validation dataset GSE100927. ROC curve analysis revealed an AUC of 0.996 (Fig. 3C), indicating superior discriminatory capacity of the 10-gene signature for distinguishing atherosclerotic pathological specimens from healthy controls, underscoring substantial clinical diagnostic utility.

**Table 2:** List of core candidate targets identified based on multidimensional screening strategies

Symbol	Full name	Function
HIF1A	Hypoxia inducible factor 1 alpha	Hypoxia response
ALOX5	Arachidonate 5-lipoxygenase	Inflammatory mediator
STAT1	Signal transducer and activator of transcription 1	Immune signaling
ITGB1	Integrin subunit beta 1	Cell adhesion
PPARG	Peroxisome proliferator-activated receptor gamma	Lipid metabolism
MMP9	Matrix metallopeptidase 9	ECM degradation
PIK3R1	Phosphoinositide-3-kinase regulatory subunit 1	PI3K pathway regulation
PRKCB	Protein kinase C beta	Signal transduction
CDK4	Cyclin dependent kinase 4	Cell cycle control
PIK3CA	Phosphatidylinositol 3-kinase catalytic subunit alpha	Cell growth

To delineate the cellular origins and spatial distribution patterns of core targets within the plaque microenvironment, the 10 core genes were projected onto single-cell UMAP embedding space for visualization (Fig. 3D). Results demonstrated non-uniform expression profiles across cellular compartments, with specific enrichment within macrophage and foam cell subsets, contrasting with relatively diminished expression in T cells, B cells, and smooth muscle cells. This cell type-specific enrichment pattern validated the efficacy of the WES selection strategy while revealing that *Perilla frutescens* likely exerts vasculoprotective effects primarily through targeted modulation of key molecular networks within intraplaque myeloid lineage cells, encompassing PPARG-mediated lipid metabolic reprogramming, MMP9-mediated extracellular matrix degradation, and HIF1A-governed hypoxic responses, thereby intercepting plaque progression and destabilization trajectories.

### Pseudotemporal dynamics and intercellular communication profiling of core targets

**Fig. 4:**
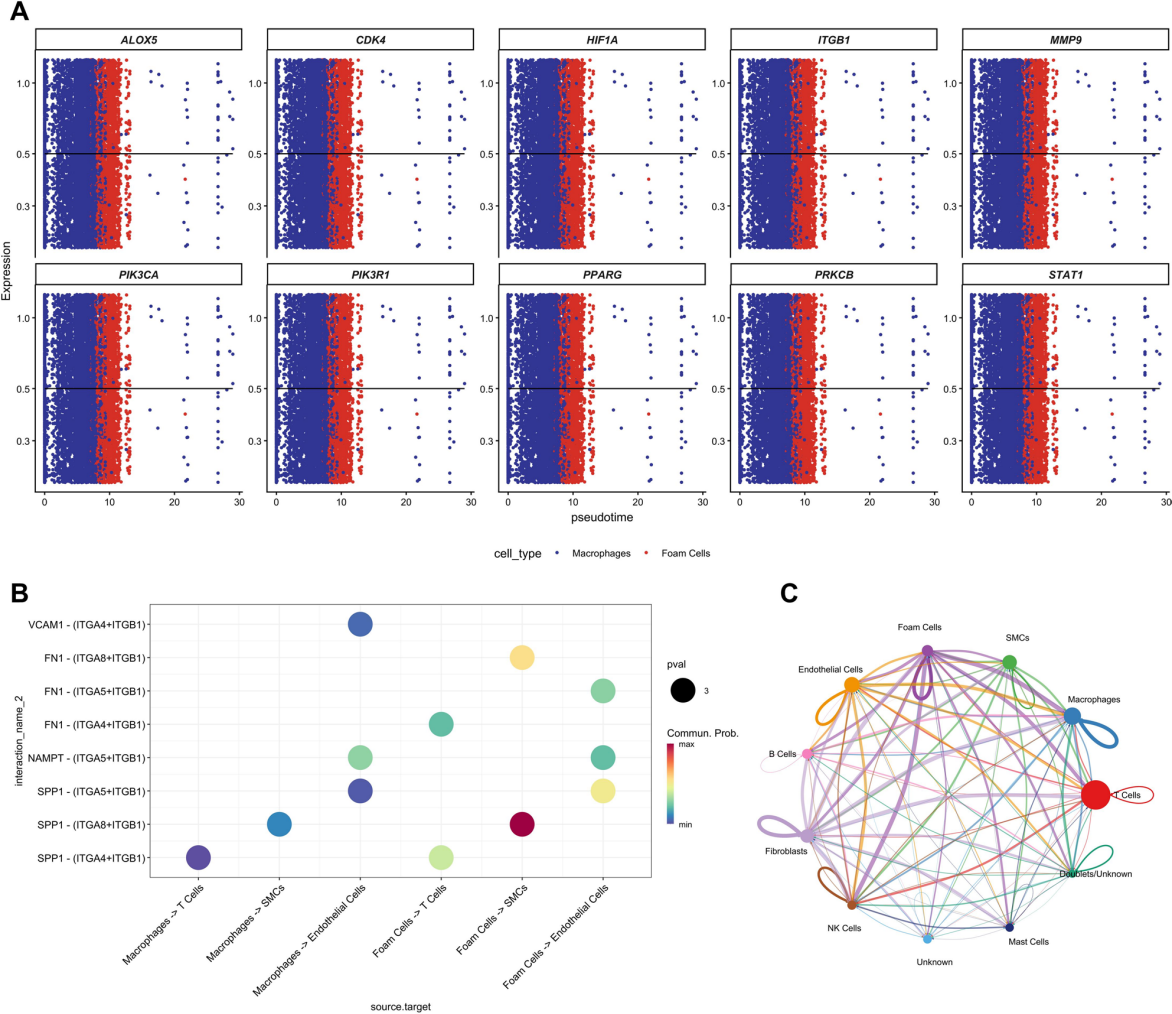
Pseudotime trajectory and cell–cell communication analysis. **A** Pseudotime expression dynamics of core targets. **B** ITGB1-based ligand-receptor interaction dot plot. **C** Overview of the intercellular communication network

Single-cell pseudotemporal trajectory reconstruction of myeloid lineage cells was performed using the Monocle3 algorithm. Trajectory reconstruction revealed continuous phenotypic evolution of intraplaque macrophages from a homeostatic root state toward a terminal foam cell state (Fig. 4A). Along this differentiation trajectory, the 10 core target genes (ALOX5, CDK4, HIF1A, ITGB1, MMP9, PIK3CA, PIK3R1, PPARG, PRKCB, STAT1) exhibited highly coordinated temporal expression dynamics, with transcriptional levels displaying sigmoidal nonlinear upregulation trajectories correlated with pseudotemporal progression. This expression architecture, exhibiting tight synchronization with cellular state transitions, indicated that these core genes not only correlate with foam cell formation but likely actively drive key pathological processes including macrophage lipid metabolic reprogramming, inflammatory transcriptional program activation, and phenotypic transformation, thereby executing pivotal regulatory functions in atherosclerotic plaque initiation and maintenance.

Furthermore, global cell–cell communication networks within the plaque microenvironment were systematically reconstructed using the CellChat toolkit to delineate functional positioning of core targets within multicellular interaction architectures. Global network topological analysis revealed intensive signaling crosstalk among macrophages, foam cells, endothelial cells, and T cells, collectively establishing a cellular communication hub orchestrating the inflammatory plaque microenvironment (Fig. 4C). Ligand–receptor interaction dissection further identified an ITGB1 (integrin β1)-centered cellular communication module (Fig. 4B). Foam cells, functioning as principal ligand-sending cells, exhibited elevated expression of pro-inflammatory/pro-fibrotic ligands including SPP1, FN1, and VCAM1; correspondingly, the receptor ITGB1 and cognate heterodimeric complexes (e.g., α4β1, α5β1 integrins) demonstrated predominant expression in endothelial cells, smooth muscle cells (SMCs), and T cells.

Ligand–receptor interaction strength quantification revealed substantial communication probabilities for SPP1–ITGB1-associated interaction pairs (including SPP1–α5β1 and SPP1–α8β1) between foam cells and smooth muscle cells/endothelial cells, implicating their involvement in mediating critical pathological processes including cell adhesion, transendothelial migration, and smooth muscle cell phenotypic switching. Collectively, these cell–cell communication network analyses suggested that *Perilla frutescens* bioactive constituents may disrupt pathological signaling between foam cells and stromal/immune cells through targeted modulation of ITGB1 receptor expression or activity, thereby attenuating inflammatory signal amplification cascades and intercepting SPP1–ITGB1 axis-mediated aberrant cellular migration and vascular microenvironmental remodeling.

### Molecular docking analysis

**Fig. 5:**
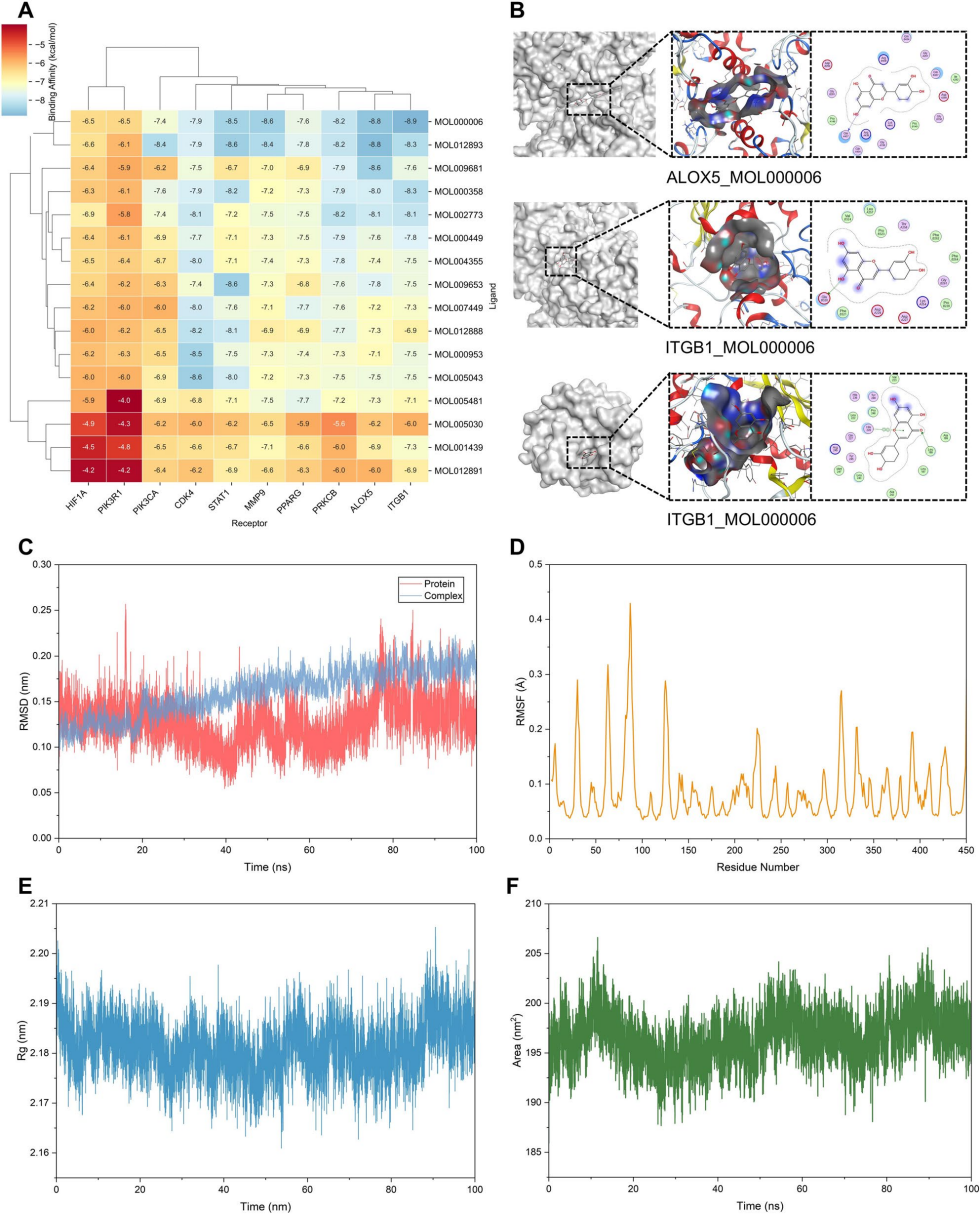
Molecular interaction and dynamic stability analysis. **A** Heatmap screening of binding affinities. **B** Molecular docking modes of Luteolin with ALOX5 and ITGB1. **C**–**F** 100-ns MD simulation analysis of the Luteolin-ITGB1 complex, including RMSD (**C**), RMSF (**D**), Rg (**E**), and SASA (**F**)

To elucidate the molecular recognition principles governing *Perilla frutescens* bioactive constituent–target interactions at atomic resolution, systematic molecular docking analyses were performed. Binding energy heatmap analysis (Fig. 5A) revealed favorable binding affinities between *Perilla frutescens* bioactive constituents and the 10 core targets (e.g., HIF1A, ITGB1), with docking scores predominantly distributed below − 5.0 kcal/mol, indicating thermodynamically favorable ligand–receptor complex formation with propensity for stable conformational association.

Notably, luteolin, the principal bioactive constituent, exhibited pronounced multi-target binding potential, demonstrating particularly favorable binding free energies with the cellular communication hub ITGB1 (− 8.9 kcal/mol), inflammatory regulatory node ALOX5 (− 8.8 kcal/mol), and extracellular matrix remodeling mediator MMP9 (− 8.6 kcal/mol). These results are indicative of robust binding affinities, suggesting pivotal contributions to the multi-target synergistic modulation of AS by *Perilla frutescens*.

Furthermore, three-dimensional docking pose analysis unveiled mechanistic details underlying molecular recognition. Within the ALOX5–luteolin complex (Fig. 5B), the ligand occupied the hydrophobic binding pocket of ALOX5, forming hydrogen bonds with the critical residue Gln437, thereby conferring conformational stabilization. Within the ITGB1–luteolin complex (Fig. 5C), the ligand occupied a putative binding pocket within the receptor extracellular domain, with hydroxyl moieties forming an extensive hydrogen bonding network with the pivotal residue Glu320, complemented by synergistic van der Waals interactions and hydrophobic effects stabilizing the binding interface. Based on the spatial conformational architecture of this binding mode, luteolin may exert competitive inhibition of endogenous ligand (e.g., SPP1) engagement with ITGB1 through steric occlusion, thereby disrupting the pathological SPP1–ITGB1 communication axis identified through cell–cell communication network analyses.

### MD simulation analysis

To assess the dynamic stability and conformational evolution characteristics of the luteolin–ITGB1 complex under quasi-physiological solvation conditions, 100-ns all-atom MD simulations were executed. RMSD trajectory analysis (Fig. 5D) revealed rapid convergence to thermodynamic equilibrium at approximately 30 ns following initial solvation relaxation. Subsequently, RMSD trajectories exhibited pronounced stability, with fluctuation amplitudes confined within a narrow 0.10–0.20 nm interval, devoid of conformational drift or ligand dissociation events, indicating preservation of protein backbone structural integrity upon ligand engagement.

RMSF analysis (Fig. 5E) quantified residue-specific local flexibility profiles. Results revealed pronounced rigidity within the ITGB1 core structural domain (RMSF < 0.20 nm), with elevated fluctuations confined to surface-exposed flexible loop regions distal to the binding site. Critically, residues within the core binding pocket exhibited minimal fluctuations, confirming that luteolin effectively stabilized the local binding conformation of ITGB1.

Rg analysis (Fig. 5F) demonstrated stable Rg values within a narrow 2.17–2.20 nm range throughout the simulation trajectory, devoid of unfolding propensity, confirming sustained maintenance of compact globular folded architecture. Additionally, solvent accessible surface area (SASA) remained stable within the 190–205 nm^2^ range (Fig. 5G), indicating invariant surface exposure characteristics within the dynamic aqueous environment, with negligible structural expansion or compaction.

Collectively, MD simulations furnished robust biophysical evidence corroborating the formation of persistent, specific, and thermodynamically stable luteolin–ITGB1 molecular complexes, establishing a theoretical foundation for developing luteolin as a candidate anti-atherosclerotic lead compound.

## Discussion

### Multi-component-multi-target-multi-pathway synergistic intervention mode of *Perilla frutescens* from the perspective of systems pharmacology

AS is essentially not a linear dysregulation of a single signaling pathway, but a systemic disease driven by the deep interweaving of lipid metabolic dysregulation and immune-inflammatory responses in spatiotemporal dimensions (Ajoolabady et al. 2024). This pathological essence determines that AS has intrinsic resistance to single-target treatment strategies. Clinical observations reveal substantial residual cardiovascular risk even among patients achieving low-density lipoprotein cholesterol (LDL-C) targets under intensive statin regimens. This phenomenon reflects the persistent activation of compensatory inflammatory pathways alongside the intrinsic robustness of pathological networks. Specifically, the homeostatic capacity of diseased systems to sustain maladaptive functional states despite localized perturbations (Kanuri et al. 2025; Tang et al. 2024).

The present investigation demonstrates that the multi-component, multi-target intervention paradigm of *Perilla frutescens* transcends mere additive pharmacological effects, instead achieving the systematic attenuation of topological robustness within AS pathological networks through synergistic perturbation of 289 disease-critical nodes, thereby precipitating a global phase transition from pathological to physiological homeostatic states. From a network pharmacology perspective, this multi-nodal synergistic intervention strategy exhibits enhanced resilience to compensatory mechanisms relative to the localized perturbations induced by single-target therapeutics, embodying a contemporary molecular-network interpretation of the holistic philosophy underpinning traditional Chinese medicine.

The pronounced enrichment of "membrane rafts" and "membrane microdomains" within GO functional analyses provides a distinctive biophysical framework for elucidating the pharmacological mechanisms of *Perilla frutescens*. Membrane rafts, characterized as cholesterol- and sphingolipid-enriched liquid-ordered microdomains within plasma membranes, constitute essential spatial platforms facilitating the assembly of functional signalosomes for inflammatory pattern recognition receptors (e.g., TLR4) and lipid scavenger receptors (e.g., CD36) (Nieto-Garai et al. 2022). Under atherogenic conditions, oxidized low-density lipoprotein (ox-LDL) binding to raft-localized CD36 receptors precipitates macrophage lipid overload, whereas pathogen-associated molecular patterns (PAMPs) amplify inflammatory cascades through the promotion of spatial clustering of TLR4/MyD88 signaling complexes within raft microdomains (Chen et al. 2022).

The phytosterols enriched in *Perilla frutescens* (e.g., β-sitosterol, stigmasterol), possessing steroid scaffolds structurally homologous to cholesterol, may intercalate into raft lipid bilayers via competitive displacement mechanisms, thereby modulating microdomain physicochemical properties encompassing membrane thickness, lipid fluidity, and membrane curvature (Zhao et al. 2025). Tang et al. (Tang et al. 2021) systematically characterized the biophysical effects of phytosterols within plant plasma membrane-mimetic lipid systems through the integration of fluorescence lifetime imaging microscopy and all-atom MD simulations. Their findings demonstrate that phytosterol incorporation induces significant reductions in membrane lipid area, elevations in bilayer thickness, enhancements in fatty acyl chain ordering, and the formation of phytosterol-enriched clusters corresponding to lipid microdomain/phase separation phenomena. This biophysical remodeling of membrane rafts disrupts the spatial clustering and functional interaction interfaces of signaling receptors, thereby imposing signal transduction blockade at the mechanistic origin, ultimately conferring membrane-targeted anti-inflammatory effects of broader spectrum than those achievable through single-receptor antagonism (Yuan et al. 2020).

KEGG pathway enrichment analysis results show that the "Lipid and atherosclerosis" pathway presents the most significant enrichment, indicating that candidate target genes are highly enriched in the functional core modules of the AS pathological network. This pathway covers the complete lipid metabolism axis from ox-LDL uptake, cholesterol esterification to reverse transport. The synergistic targeting of multiple critical nodes within this pathway by *Perilla frutescens* bioactive constituents may disrupt the lipid accumulation–inflammation activation positive feedback loop inherent to foam cell formation through a bidirectional regulatory paradigm of upstream flux restriction coupled with downstream efflux enhancement (Kong et al. 2022). *Perilla frutescens* harbors an extensive repertoire of bioactive constituents (including α-linolenic acid, flavonoids, and phenolic acids) that exert synergistic actions upon multiple critical processes encompassing lipid uptake, cholesterol esterification, reverse cholesterol transport, oxidative stress mitigation, and inflammatory response attenuation (Hou et al. 2022). Evidence from animal models and cellular experiments demonstrates that *Perilla frutescens* extracts downregulate the expression of ox-LDL scavenger receptors (CD36, LOX-1), suppress foam cell formation, facilitate cholesterol efflux through the upregulation of ABCA1 and SR-B1 expression, and substantially attenuate the secretion of pro-inflammatory cytokines (e.g., IL-1β, MCP-1) (Pothinam et al. 2025).

Impaired efferocytosis precipitates secondary necrosis of apoptotic cells and progressive expansion of necrotic cores, processes intimately associated with vulnerable plaque formation and the incidence of acute coronary syndrome (ACS) (Morrissey et al. 2020). *Perilla frutescens* may restore intraplaque efferocytic efficiency through the upregulation of phagocytic receptors (e.g., MerTK), the activation of peroxisome proliferator-activated receptor gamma (PPARγ)-dependent bridging molecule secretion, and the suppression of the inhibitory CD47–SIRPα axis. The restoration of efferocytic capacity not only diminishes necrotic core dimensions but also initiates active inflammation resolution programs through the liberation of specialized pro-resolving mediators (SPMs), including lipoxins and resolvins (Filep 2022).

The pronounced enrichment of the neutrophil extracellular trap (NET) formation (NETosis) pathway suggests the potential modulatory capacity of Perilla frutescens in immunothrombosis. NETs exert pathogenic actions across multiple stages of AS progression: during early phases, histones impose cytotoxic injury upon vascular endothelium; throughout disease advancement, NETs function as damage-associated molecular patterns (DAMPs) activating NLRP3 inflammasomes to amplify inflammatory responses; and during acute events, NETs serve as thrombogenic scaffolds capturing platelets and coagulation factors (Döring et al. 2017). Luteolin, a cardinal bioactive constituent of *Perilla frutescens*, demonstrates established capacity for inhibiting NADPH oxidase (NOX2) activity. Given that NOX2-mediated reactive oxygen species (ROS) generation constitutes a critical initiating determinant of NETosis, these findings suggest that *Perilla frutescens* may suppress NET formation at mechanistic origins (Xia et al. 2014).

Furthermore, the substantial enrichment of the advanced glycation end product (AGE)–receptor for AGE (RAGE) signaling pathway suggests distinctive therapeutic potential for *Perilla frutescens* in patients with concomitant diabetes mellitus and AS. The hyperactivation of the AGE–RAGE axis perpetuates oxidative stress amplification and inflammatory signal cascades via nuclear factor-κB (NF-κB) pathways, constituting a pivotal pathogenic mechanism underlying accelerated AS progression in diabetic populations (B. Wang et al. 2025a, b).

### Molecular analysis of foam cell differentiation trajectory and core target identification at single-cell resolution

Conventional transcriptomic investigations relying upon bulk RNA sequencing (bulk RNA-seq) frequently obscure the profound cellular heterogeneity within atherosclerotic plaques through population-averaging effects, thereby precluding the precise identification of critical molecular events (Tzec‐Interián et al. 2025). The present study reconstructed the comprehensive cellular atlas of human carotid atherosclerotic plaques through the integration of high-resolution single-cell RNA sequencing (scRNA-seq) data, thereby achieving precise discrimination between foam cells and classical macrophage subpopulations. Building upon this foundation, the implementation of a dual-algorithm feature selection framework integrating least absolute shrinkage and selection operator (LASSO) regression and random forest methodologies enabled the precise identification of a 10-gene signature module centered upon HIF1A, PPARG, and ALOX5.

For instance, Xu et al. (Xu et al. 2022) integrated multiple Gene Expression Omnibus (GEO) microarray datasets, identifying 611 AS-associated differentially expressed genes through differential expression analyses, subsequently employing multiple machine learning algorithms (LASSO, random forest) for key gene selection, and validating findings across external human and murine specimens. Their investigation ultimately proposed a diagnostic gene pair comprising DHRS9 and PTPRJ, which exhibited robust discriminatory capacity between AS and control samples and demonstrated strong associations with diverse immune cell infiltration patterns. Ban et al. (Ban et al. 2024) performed weighted gene co-expression network analysis (WGCNA) and differential expression profiling on AS and ischemic stroke (IS) datasets, constructing shared differentially expressed gene networks and identifying ATF3, CCL3, CCL4, JUNB, KRAS, and ZC3H12A as shared hub genes potentially participating in the pathological processes underlying both AS and IS, with subsequent validation of expression trends via quantitative real-time PCR (qPCR) analysis of clinical specimens. This gene module represents not a stochastic assemblage but rather a constellation profoundly reflecting the immunometabolic reprogramming signature inherent to foam cell formation. Pseudotemporal trajectory reconstruction analyses confirmed that the expression abundance of this gene module exhibits highly coordinated sigmoidal upregulation dynamics along pathological differentiation trajectories, strongly implicating these core genes not as passive markers of foam cell states but rather as cardinal regulatory determinants actively orchestrating phenotypic transitions.

The pronounced upregulation of HIF1A unveils the distinctive hypoxic microenvironmental signature characterizing plaque core regions. With progressive plaque expansion, oxygen diffusion limitations precipitate substantial reductions in local oxygen tension, thereby triggering HIF1A-dependent metabolic reprogramming characterized by the metabolic transition from oxidative phosphorylation to glycolysis (the Warburg effect) (Qiu et al. 2023). While this metabolic shift sustains cellular bioenergetic homeostasis under hypoxic conditions in the short term, it precipitates persistent lactate accumulation. Lactate, through the activation of G protein-coupled receptor 81 (GPR81), suppresses cyclic adenosine monophosphate (cAMP) signaling cascades, thereby further attenuating anti-inflammatory programs and exacerbating macrophage polarization toward M1-type pro-inflammatory phenotypes, ultimately establishing a self-perpetuating hypoxia–metabolism–inflammation vicious cycle.

As a cardinal member of the lipid-sensing nuclear receptor superfamily, PPARγ facilitates reverse cholesterol transport (RCT) under physiological conditions through the transcriptional activation of lipid efflux transporters (e.g., ABCA1/ABCG1), thereby conferring atheroprotective effects (Zhang et al. 2024). However, under conditions of sustained intraplaque lipid overload, PPARγ function may undergo pathological “hijacking”: ligand activation upregulates fatty acid translocase expression (e.g., CD36), paradoxically exacerbating dysregulated ox-LDL uptake; concurrently, elevated oxidized lipid concentrations may impair PPARγ–coactivator interactions through covalent modifications, thereby attenuating its transcriptional activation capacity. This double-edged sword phenomenon of PPARγ functionality partially explains the failure of PPARγ agonist monotherapy to substantially improve clinical outcomes in AS patients (Riccioni et al. 2009). LTB4 exerts potent chemotactic actions via leukotriene B4 receptor 1 (BLT1), orchestrating the recruitment of neutrophils and monocytes into atherosclerotic plaques, whereas CysLTs, acting through CysLT1 receptors, promote vascular smooth muscle cell contraction and endothelial permeability elevations. The sustained generation of ALOX5 metabolites and inflammatory cell infiltration establish a positive feedback loop, constituting a pivotal molecular mechanism underlying the recalcitrance of chronic plaque inflammation to spontaneous resolution (Kotlyarov & Kotlyarova 2022).

Furthermore, these 10 core gene targets demonstrated exceptional diagnostic performance (AUC = 0.996) within independent external validation cohorts. These findings not only substantiate the robustness and reproducibility of the feature selection framework but also underscore the substantial clinical translational potential of these core molecules as liquid biopsy biomarkers for AS.

### Analysis of cell–cell communication network and elucidation of ITGB1 hub function

Atherosclerotic plaques constitute not static cellular aggregations but rather highly dynamic cellular ecosystems sustained by intricate intercellular communication networks (Raju et al. 2025). Diverse cellular populations within plaques establish dense information exchange networks through ligand–receptor interactions, extracellular vesicle (EV) trafficking, and metabolite signaling. These multicellular coordination patterns collectively orchestrate plaque inflammatory microenvironmental characteristics and structural integrity (Raju et al. 2024). The present investigation employed CellChat computational frameworks to systematically dissect the global intercellular communication landscape within plaques, thereby unveiling the molecular mechanisms through which *Perilla frutescens* exerts therapeutic effects via targeted disruption of the SPP1–ITGB1 signaling axis. Secreted phosphoprotein 1 (SPP1), predominantly expressed by discrete macrophage subpopulations, constitutes a pivotal signaling hub orchestrating AS progression and plaque destabilization (Li et al. 2025). SPP1 promotes fibrotic responses within fibro-progenitor cells through integrin receptor engagement (encompassing ITGB1, ITGAV/ITGB5). Early functional investigations demonstrate that the SPP1–integrin signaling axis drives vascular smooth muscle cell (VSMC) migration and phenotypic switching, processes intimately associated with intimal thickening and foam cell formation (Huang et al. 2024).

Ligand–receptor interaction analyses reveal that SPP1 (osteopontin, OPN), abundantly expressed and secreted by foam cells, represents the predominant ligand activating integrin β1 (ITGB1) on vascular wall cells (endothelial cells, smooth muscle cells) and infiltrating immune cells (Yim et al. 2022). ITGB1, as the cardinal β subunit within the integrin superfamily, heterodimerizes with diverse α subunits (e.g., α4β1, α5β1, α8β1), exerting nodal regulatory functions in cellular adhesion, migration, and mechanotransduction through bidirectional "outside-in" and "inside-out" signaling mechanisms (Su et al. 2024).

SPP1 engagement with endothelial α4β1/α5β1 integrins activates focal adhesion kinase (FAK)–Src signaling cascades, precipitating tyrosine phosphorylation and subsequent endocytic degradation of vascular endothelial cadherin (VE-cadherin), thereby compromising adherens junction integrity (Fei et al. 2025). The attenuation of endothelial barrier function precipitates elevations in vascular permeability, thereby facilitating transendothelial migration (TEM) of circulating monocytes and sustaining the replenishment of intraplaque inflammatory cell reservoirs (Dalal et al. 2019).

Under physiological conditions, vascular smooth muscle cells (VSMCs) manifest a contractile phenotype essential for the maintenance of vascular tone and structural integrity (Cao et al. 2022). ITGB1, functioning as a cardinal mechanosensor, transduces ECM mechanical cues into intracellular biochemical signals. ITGB1 signaling cascades drive VSMC phenotypic switching toward synthetic states through the activation of downstream phosphoinositide 3-kinase (PI3K)/protein kinase B (Akt) and mitogen-activated protein kinase (MAPK)/extracellular signal-regulated kinase (ERK) pathways, manifested by the downregulation of contractile proteins (α-smooth muscle actin [α-SMA], smooth muscle myosin heavy chain [SM-MHC]) alongside the upregulation of matrix synthesis proteins (collagen, proteoglycans) and matrix metalloproteinases (MMPs) (Wu et al. 2025). The excessive proliferation and enhanced apoptosis of synthetic VSMCs ultimately compromise plaque structural stability, thereby elevating plaque rupture risk.

Predicated upon these mechanisms, *Perilla frutescens* bioactive constituents may interrupt the transmission of pathological signals from foam cells to the microenvironment through targeted disruption of SPP1–ITGB1 interactions at the intercellular communication level, thereby achieving: restoration of endothelial barrier functionality to attenuate inflammatory cell infiltration; preservation of VSMC contractile phenotypes to stabilize plaque architecture; and suppression of excessive MMP activation to prevent fibrous cap rupture. This intervention paradigm predicated upon the disruption of pathological intercellular communication furnishes a conceptually innovative therapeutic framework for AS that transcends conventional intracellular signaling pathway targeting.

### Multi-target binding characteristics of luteolin and kinetic stability of ITGB1 complex

Molecular docking and MD simulations furnished atomic-resolution structural and kinetic evidence characterizing interactions between *Perilla frutescens* bioactive constituents and core targets. Binding energy heatmap analyses reveal favorable binding affinities of 16 *Perilla frutescens* constituents toward the 10 core targets, indicating thermodynamic spontaneity of ligand–receptor complex formation.

Within the *Perilla frutescens* bioactive constituent repertoire, luteolin exhibits pronounced multi-target binding potential. As a prototypical flavonoid, the molecular architecture of luteolin, encompassing A/B dual aromatic ring systems, C-ring C2–C3 unsaturation, and multiple phenolic hydroxyl moieties, confers the structural foundation for establishing stable non-covalent interactions with diverse protein targets. Docking analyses demonstrate that luteolin exhibits favorable binding free energies toward the intercellular communication hub ITGB1 (− 8.9 kcal/mol), the inflammatory rate-limiting enzyme ALOX5 (− 8.8 kcal/mol), and the extracellular matrix degradation effector MMP9 (− 8.6 kcal/mol).

Three-dimensional conformational analyses unveil the molecular recognition mechanisms underlying luteolin–target interactions. Within the ALOX5–luteolin complex, the ligand intercalates into the hydrophobic binding cavity of the catalytic domain, establishing stable interactions with regions proximal to the non-heme iron active site; the A-ring phenolic hydroxyl forms a hydrogen bond with the critical residue Gln437, thereby anchoring the binding conformation. The spatial occupancy of the active site by luteolin may obstruct leukotriene biosynthetic pathways through competitive inhibition of substrate access (Ren et al. 2021). Within the ITGB1–luteolin complex, the ligand occupies the ligand-binding pocket of the extracellular I-like domain of ITGB1; the C4′-hydroxyl forms a hydrogen bond with the carboxyl side chain of the critical residue Glu320, while the B-ring engages in π-alkyl interactions with hydrophobic residues lining the pocket interior (De Aguiar et al. 2025). This binding site coincides precisely with the recognition interface for endogenous ligands such as SPP1, suggesting that luteolin may competitively abrogate pathological SPP1–ITGB1 interactions through steric occlusion (Zhou et al. 2024).

While molecular docking furnishes static structural snapshots of ligand–receptor interactions, proteins undergo continuous conformational fluctuations under physiological conditions. The capacity of ligands to sustain stable binding within dynamic aqueous environments directly governs the duration and magnitude of pharmacological activity (Fu et al. 2018). This property assumes particular significance for ITGB1, given that as a mechanotransduction receptor, its functionality critically depends upon dynamic transitions between bent low-affinity and extended high-affinity conformational states. The present investigation systematically assessed the spatiotemporal dynamic evolution of the luteolin–ITGB1 complex through 100-ns all-atom MD simulations.

RMSD trajectory analyses reveal rapid convergence of the complex to thermodynamic equilibrium at approximately 30 ns; subsequently, fluctuation amplitudes remained within a narrow 0.10–0.20 nm range, with no observable ligand dissociation or substantial conformational drift throughout the simulation. This rapid equilibration characteristic suggests that luteolin efficiently accommodates the receptor binding pocket microenvironment, indicating potential for prompt in vivo onset of action.

The local conformational stabilization effects unveiled through RMSF analyses constitute a cardinal finding of the present investigation. Core residues within the ligand-binding pocket, most notably Glu320 and adjacent hydrophobic residue clusters, exhibit substantially diminished fluctuation amplitudes (RMSF < 0.20 nm), whereas regions of elevated fluctuation remain confined to surface-exposed flexible loops distal to the binding interface. This local rigidification may help luteolin stabilize ITGB1 through binding pocket occupancy and multivalent non-covalent interactions, thereby reducing receptor responsiveness to endogenous ligands such as SPP1 or mechanical stimuli. Given that ITGB1 signal transduction relies upon ligand-induced conformational changes (outside-in signaling), this conformational locking effect may fundamentally abrogate downstream FAK/Src signaling cascades mediated by the SPP1–ITGB1 axis (Su et al. 2024).

From a medicinal chemistry perspective, ligand-induced binding pocket rigidification diminishes conformational entropy at the binding interface, thereby elevating the energy barrier for ligand dissociation through enthalpy–entropy compensation mechanisms (Stank et al. 2016). This kinetics-driven pharmacological advantage assumes particular importance for chronic pathologies such as AS requiring sustained therapeutic intervention (Wang et al. 2020). This conformational locking mechanism significantly prolongs the receptor residence time of luteolin, thereby allowing the ligand to sustain target occupancy and functional blockade even under conditions of declining plasma concentrations.

The stability of the Rg (2.17–2.20 nm) and solvent-accessible SASA (190–205 nm^2^) throughout the simulation trajectory indicates that ITGB1 maintains a compact, globular folded conformation without structural unfolding or long-range allosteric propagation. This binding mode substantiates the mechanistic hypothesis that luteolin functions as an orthosteric competitive inhibitor of the SPP1–ITGB1 axis, disrupting pathological intercellular communication through direct occupancy of the ligand recognition interface rather than indirect allosteric modulation. Integration with the aforementioned CellChat intercellular communication network analyses suggests that luteolin targeted intervention upon ITGB1 may sever pro-inflammatory and pro-remodeling signals emanating from foam cells into the vascular microenvironment at the intercellular communication level, thereby conferring multifaceted protective effects encompassing endothelial barrier restoration, VSMC contractile phenotype preservation, and extracellular matrix degradation suppression (Wu et al. 2018).

### Limitations and future perspectives

Although this study used an integrated computational framework to explore the anti-atherosclerotic mechanisms of Perilla frutescens, several limitations remain. The bioactive constituents and therapeutic targets were mainly predicted from public databases, so database bias, incomplete annotation, and false-positive results cannot be excluded. Although the core targets were supported by single-cell transcriptomic analysis and external validation, these associations do not fully prove causal regulatory effects. The machine learning results may also be affected by sample size, biological heterogeneity, data preprocessing, and batch effects.

Molecular docking and molecular dynamics simulations suggested stable luteolin–ITGB1 binding, but these methods cannot fully reflect pharmacokinetic behavior or intracellular complexity. In addition, this study lacks direct cellular and animal experimental validation. Future studies should verify the effects of luteolin and other Perilla frutescens-derived constituents on the SPP1–ITGB1 axis using foam cell formation assays, endothelial adhesion assays, ITGB1 knockdown or overexpression models, and atherosclerosis animal models.

## Conclusion

In summary, the present investigation establishes a multiscale computational analytical framework integrating single-cell transcriptomics, machine learning algorithms, and MD simulations to systematically elucidate the multi-target synergistic mechanisms underlying the therapeutic intervention of *Perilla frutescens* frutescens seed in AS. The investigation identifies a 10-gene core signature module encompassing HIF1A, PPARG, and ITGB1, which not only demonstrates exceptional diagnostic performance within independent clinical validation cohorts (AUC = 0.996) but also profoundly captures the molecular signatures of immunometabolic reprogramming accompanying macrophage-to-foam cell differentiation. More critically, the study furnishes atomic-resolution biophysical evidence substantiating that luteolin, a cardinal bioactive constituent of *Perilla frutescens*, engages integrin β1 (ITGB1) with high specificity through conformational locking mechanisms, thereby spatially disrupting the foam cell-driven SPP1–ITGB1 pro-inflammatory communication axis. Collectively, these findings suggest that the targeted disruption of pathological cell adhesion receptors and their mediated signal crosstalk constitutes a highly promising innovative therapeutic strategy for addressing RIR in AS. While the present investigation primarily employs computational biology and molecular simulation strategies, future studies warrant systematic validation of the pharmacokinetic/pharmacodynamic (PK/PD) profiles, in vivo targeting efficiency, and long-term therapeutic efficacy of luteolin–ITGB1 interactions within animal models and clinical trials, alongside comprehensive exploration of its clinical translational potential as a precision therapeutic candidate for AS.

## Data Availability

Target intersection analysis and Venn diagram visualization were performed in R (v4.3.2) with the ggVennDiagram package (v1.2.2). Subsequent PPI network construction, functional enrichment and single-cell transcriptomic analysis were carried out via standard bioinformatics pipelines. All public database resources are freely accessible via the listed URLs. Generated processed datasets are available upon reasonable request to the corresponding author for research replication.

## Abbreviations

ADME: Absorption, distribution, metabolism, and excretion
AGE: Advanced glycation end product
AGE–RAGE: Advanced glycation end product–receptor for advanced glycation end product
AS: Atherosclerosis
AUC: Area under the curve
BH: Benjamini–Hochberg
BP: Biological process
CC: Cellular component
CD36: Cluster of differentiation 36
CI: Confidence interval
DEG: Differentially expressed gene
DL: Drug-likeness
ECM: Extracellular matrix
FAK: Focal adhesion kinase
FDR: False discovery rate
GEO: Gene expression omnibus
GO: Gene Ontology
HIF1A: Hypoxia-inducible factor 1 subunit alpha
HVG: Highly variable gene
ITGB1: Integrin subunit beta 1
KEGG: Kyoto encyclopedia of genes and genomes
LASSO: Least absolute shrinkage and selection operator
LDL-C: Low-density lipoprotein cholesterol
MD: Molecular dynamics
MF: Molecular function
ML: Machine learning
NET: Neutrophil extracellular trap
NF-κB: Nuclear factor kappa B
NK: Natural killer
OB: Oral bioavailability
OMIM: Online Mendelian inheritance in man
ox-LDL: Oxidized low-density lipoprotein
PCA: Principal component analysis
PCSK9: Proprotein convertase subtilisin/kexin type 9
PDB: Protein data bank
PPI: Protein–protein interaction
PPARG: Peroxisome proliferator-activated receptor gamma
QC: Quality control
RF: Random forest
Rg: Radius of gyration
RIR: Residual inflammatory risk
RMSD: Root mean square deviation
RMSF: Root mean square fluctuation
ROC: Receiver operating characteristic
ROS: Reactive oxygen species
SASA: Solvent accessible surface area
scRNA-seq: Single-cell RNA sequencing
SEA: Similarity ensemble approach
SMC: Smooth muscle cell
SPP1: Secreted phosphoprotein 1
TCMSP: Traditional Chinese Medicine Systems Pharmacology
UMAP: Uniform manifold approximation and projection
VSMC: Vascular smooth muscle cell
WES: Weighted expression score
